# Overexpression of miR‐19a and miR‐20a in iPS‐MSCs preserves renal function of chronic kidney disease with acute ischaemia‐reperfusion injury in rat

**DOI:** 10.1111/jcmm.16613

**Published:** 2021-06-23

**Authors:** Mel S. Lee, Hon‐Kan Yip, Chih‐Chao Yang, John Y. Chiang, Tien‐Hung Huang, Yi‐Chen Li, Kuan‐Hung Chen, Pei‐Hsun Sung

**Affiliations:** ^1^ Department of Orthopedics Kaohsiung Chang Gung Memorial Hospital and Chang Gung University College of Medicine Kaohsiung Taiwan; ^2^ Division of Cardiology Department of Internal Medicine Kaohsiung Chang Gung Memorial Hospital and Chang Gung University College of Medicine Kaohsiung Taiwan; ^3^ Institute for Translational Research in Biomedicine Kaohsiung Chang Gung Memorial Hospital Kaohsiung Taiwan; ^4^ Center for Shockwave Medicine and Tissue Engineering Kaohsiung Chang Gung Memorial Hospital Kaohsiung Taiwan; ^5^ Department of Medical Research China Medical University Hospital China Medical University Taichung Taiwan; ^6^ Department of Nursing Asia University Taichung Taiwan; ^7^ Division of Cardiology Department of Internal Medicine Xiamen Chang Gung Hospital Xiamen China; ^8^ Division of Nephrology Department of Internal Medicine Kaohsiung Chang Gung Memorial Hospital and Chang Gung University College of Medicine Kaohsiung Taiwan; ^9^ Department of Computer Science and Engineering National Sun Yat‐Sen University Kaohsiung Taiwan; ^10^ Department of Anesthesiology Kaohsiung Chang Gung Memorial Hospital and Chang Gung University College of Medicine Kaohsiung Taiwan

**Keywords:** chronic kidney disease, fibrosis, inflammation, iPS‐MSCs, ischaemia‐reperfusion injury, microRNAs, oxidative stress

## Abstract

This study tested the hypothesis that therapy with double overexpression of miR‐19a‐3p and miR‐20a‐5p (miR^DOE^) to human inducible pluripotent stem cell–derived mesenchymal stem cells (iPS‐MSCs) was superior to iPS‐MSCs alone for preserving renal function in rat with pre‐existing chronic kidney disease (CKD), followed by ischaemia‐reperfusion (IR) injury. In vitro study demonstrated that the protein expressions of oxidative stress (NOX‐1/NOX‐2/NOX4/oxidized protein/p22phox), inflammatory downstream signalling (TLR2&4/MyD88/TRAF6/IKK‐ß/p‐NFκB/IL‐1ß/IL‐6/MMP‐9) and cell apoptosis/death signalling (cleaved caspase‐3/mitochondrial Bax/p‐ERKs/p‐JNK/p‐p38) at time‐points of 24‐hour/48‐hour cell cultures were significantly increased in p‐Cresol‐treated NRK‐52E cells than in the control that was significantly reversed by miR‐19a‐3p‐transfected iPS‐MSC (all *P* < .001). Animals were categorized into group 1 (sham‐operated control), group 2 (CKD‐IR), group 3 (CKD‐IR + oligo‐miR^DOE^ of iPS‐MSCs/6.0 ×10^5^/intra‐renal artery transfusion/3 hours after IR procedure), group 4 (CKD‐IR + iPS‐MSCs) and group 5 (CKD‐IR + miR^DOE^ of iPS‐MSCs/6.0 ×10^5/^intra‐renal artery transfusion/3 hour after IR procedure). By day 35, the creatinine/BUN levels were lowest in group 1, highest in group 2 and significantly lower in group 5 than in groups 3 and 4 (all *P* < .0001) but they showed no difference between the latter two groups. The protein expressions of oxidative stress, inflammatory downstream signalling and cell apoptosis/death signalling exhibited an identical pattern of creatinine level among the five groups (all *P* < .00001). Also, the microscopic findings demonstrated that the kidney injury score/fibrotic area/number of inflammatory cells (CD14+/CD68+) exhibited an identical pattern of creatine level (all *P* < .0001). The miR^DOE^ of iPS‐MSCs was superior to iPS‐MSCs for preserving the residual kidney function and architecture in CKD‐IR rat.

## 1 INTRODUCTION

Acute kidney injury (AKI) with and without pre‐existing chronic kidney disease (CKD), which is common in hospitalized patients, includes a group of clinical syndromes that primarily manifest as a rapid decline in renal function in association with the accumulation of metabolic waste.^1^, ^2^ Previous study has shown that about 17 million hospital admissions per year in the United States are complicated by AKI, resulting in additional costs up to $10 billion to the healthcare system.^3^ Additionally, AKI has been estimated to occur in approximately 10%‐15% of patients admitted to hospital, while its incidence in intensive care unit has been found to be in more than 50% of patients.^4^, ^5^ This situation, therefore, warrants the development of new therapeutic modalities for improving the kidney function after AKI,^6^, ^7^, ^8^ especially in pre‐existing CKD setting.

Summarizing from the literatures surveyed, we can find that the CKD, with variously divergent entities of known and unknown causal aetiologies clearly identified,^9^, ^10^, ^11^, ^12^, ^13^, ^14^ is still the major and rapidly growing contributor to healthcare burden worldwide.^14^, ^15^, ^16^ Abundant data from clinical observational studies have revealed that CKD incurred high morbidity and mortality in hospitalized patients, especially in those CKD patients with co‐existing cardiovascular disease (ie cardiorenal syndrome).^14^, ^17^, ^18^, ^19^, ^20^, ^21^, ^22^

Intriguingly, despite the state‐of‐the‐art pharmaceutical strategies, such as the uses of angiotensin‐converting enzyme inhibitor (ACEI), angiotensin II type I receptor blockade (ARB), and direct renin inhibitor (DRI), as well as good education, and renewed guideline for CKD precision management, progressive deterioration of residual renal function in the setting of CKD is commonly encountered, as a subsequence of leading to the adverse development of end‐stage renal disease (ESRD).^23^, ^24^, ^25^, ^26^, ^27^, ^28^ Thus, current treatment of CKD is regrettably left much to be desired.

Intriguingly, plentiful studies^29^, ^30^, ^31^, ^32^, ^33^, ^34^ have demonstrated that mesenchymal stem cell (MSC) and endothelial progenitor cell (EPC) treatments for CKD are safe and efficacious maintenance of residual renal function in CKD setting. Additionally, studies have recently further revealed that human induced pluripotent stem cell (iPSC)–derived MSCs (ie iPS‐MSCs) exhibit multiple paracrine actions (ie including angiogenesis, immunomodulatory and anti‐inflammatory factors) for organ repair and regeneration due to strong capacities of self‐renewal and differentiation into most somatic cell lineages.^35^, ^36^ Furthermore, our previous study^37^ also demonstrated that iPSC‐derived MSC therapy efficaciously safeguarded the rodent renal function and kidney architecture from acute ischaemia‐reperfusion (IR) injury.

Recently, our phase I clinical trial demonstrated that intra‐renal artery administration of autologous EPCs (ie CD34+ cell therapy) to CKD patients was safe and retained the renal function in a stable state at one‐year follow‐up.^33^ In this study, we also found that the gene expressions of miR‐374a‐5p, miR‐19a‐3p, miR‐106b‐5p, miR‐26b‐5p and miR‐20a‐5p in peripheral blood mononuclear cells, five anti‐apoptotic miRNAs, were significantly lower in CKD patients than in healthy individuals.^33^ These findings^33^, ^35^, ^36^ raised the hypothesis that transfected miR‐19a‐3p and miR‐20a‐5p (ie double miRNA overexpression) to human iPS‐MSCs were superior to iPS‐MSCs only for preserving the residual renal function in rat CKD.

## 2 MATERIALS AND METHODS

### 2.1 Ethics

All animal procedures were approved by the Institute of Animal Care and Use Committee at Kaohsiung Chang Gung Memorial Hospital (Affidavit of Approval of Animal Use Protocol No. 2017102401) and performed in accordance with the Guide for the Care and Use of Laboratory Animals.

Animals were housed in an Association for Assessment and Accreditation of Laboratory Animal Care International (AAALAC; Frederick, MD, USA)–approved animal facility in our hospital with controlled temperature and light cycles (24°C and 12/12‐hour light cycle).

### 2.2 miR‐19a‐3p and miR‐20a‐5p were candidates for double overexpression (miR^DOE^) in iPS‐MSCs and treatment of CKD animals

We have evaluated that miR‐19a‐3p and miR‐20a‐5p were the two most suitable candidates among the five miRNAs (ie miR‐374a‐5p/miR‐19a‐3p/miR‐106b‐5p/miR‐26b‐5p/miR‐20a‐5p) to be overexpressed (ie transfection) for the purpose of treatment of CKD + IR animals. In detail, transfections of miR‐19a‐3p and miR‐20a‐5p mimics efficiently increased miRNA expressions and further decreased related gene expressions. Transfections of mimics (25 nmol/L) were carried out with TransIT‐X2 Dynamic Delivery System (Mirus), according to the manufacturer's instruction (ie [the product model of miRNA mimics: Syn‐hsa‐miR‐19a‐3p (Cat. MSY0000073, Thermo Fisher Scientific) and Syn‐hsa‐miR‐20a‐5p (Cat. MSY0000075, Thermo Fisher Scientific)]. The iPS‐MSCs were identified >80% confluence on the day of transfection. TransIT‐X2 reagent was mixed with miRNA mimics for 25 minutes at room temperature. The miRNA mimics‐containing complexes were further distributed into cells. Two days later, expressions of miRNAs and related genes were validated by the real‐time qPCR assay (Figure S1).

### 2.3 Creation of animal model of CKD

The procedure and protocol of CKD induction have been described in detail in our previous report.^29^, ^31^ Pathogen‐free, adult male Sprague‐Dawley (SD) rats (n = 40) weighing 320‐350 g (Charles River Technology, BioLASCO Taiwan Co. Ltd.) were anaesthetized by inhalational 2.0% isoflurane and placed supine on a warming pad at 37°C for midline laparotomies. The shame‐operated control (SC) rats received laparotomy only, while CKD was induced in all animals of the CKD groups by right nephrectomy plus arterial ligation of the upper two‐third (upper and middle poles) blood supplies of the left kidney, leaving the lower third (lower pole) kidney with normal blood supply. This model allowed preservation of a limited amount of functioning renal parenchyma and simulation of CKD.

### 2.4 Animal grouping, procedure and protocol for acute kidney ischaemia‐reperfusion (IR) injury in pre‐existing CKD and therapeutic strategy

The procedure and protocol of acute kidney IR injury have been previously described.^37^, ^38^ Briefly, by day 28 after CKD induction, those of CKD animals were again anaesthetized by inhalational 2.0% isoflurane and placed supine on a warming pad at 37°C for midline laparotomies. SC animals underwent laparotomy only, while acute kidney IR injury of left kidney was induced in all animals in groups 2 to 5 by clamping the renal pedicles for 50 minutes using non‐crushing vascular clips.

Followed by IR procedure, the animals were divided into group 1 [sham‐operated control (SC)], group 2 (CKD + IR), group 3 (CKD + IR + oligo‐miR^DOE^ of iPS‐MSCs by intra‐renal artery transfusion 3 hours after IR procedure), group 4 [CKD + IR + iPS‐MSCs (6.0 × 10^5^ cells) by intra‐renal artery transfusion 3 hours after IR procedure] and group 5 [CKD + IR + miR^DOE^ of iPS‐MSCs (6.0 × 10^5^ cells) by intra‐renal artery transfusion 3 hours after IR procedure], respectively.

The animals in each group were killed and kidney specimens collected for individual study by day 7 after the acute kidney IR induction, that is the total study period was 35 days (CKD was 28 days + 7 days after IR procedure = 35 days). The time‐point of iPS‐MSC administration to the animals at 3 hours after AK‐IR was based on our recent reports.^37^, ^39^ Additionally, the dosage and the route of intra‐renal artery administration were based on our previous report^32^ with minimal modification. Furthermore, based on the concept that the intravenous administration of stem cell will frequently be trapped into the lung parenchyma, the purpose of intra‐renal artery administration of cells in the present study was to make sure the cells were precisely into renal circulation rather were retained in the lung parenchyma for an achievement of great therapeutic effect.

### 2.5 Procedure and protocol of cell culture for differentiation of human iPS into iPS‐MSCs

The procedure and protocol of human iPSC culture for differentiation into iPS‐MSCs were as per the manufacturer's instructions and have been described in our previous report.^37^, ^39^ In detail, by day 1, human iPSCs (mTeSR™1; StemCell, #28315) were first washed by 5 mL PBS, followed by 2 mL Accutase (Gibco, #A1110501; Accutase: PBS = 1:1); the incubator reaction was continuous for 1 minute. Additionally, 2 mL KO DMEM/F12 (Gibco, #12660012) was added and the cells were collected in 15‐mL centrifuge tubes for 5‐minute centrifugation (200 *g*). The cells were then cultured in a 10‐cm dish for 24 hours in mTeSR™1 culture medium. By day 2, the cells (mTeSR™1) were collected and washed with 5 mL PBS. STEMdiff™‐ACF Mesenchymal Induction Medium (StemCell, #05241) was added and the incubator culture was continued for 24 hours. The STEMdiff™‐ACF Mesenchymal Induction Medium was exchanged once/day from days 1 to 3. This procedure was repeated on days 3‐6. On days 7‐21, the procedure was repeated but the culture medium was refreshed every 3 days. As the standard method of iPS‐MSC culture, the descriptions were similar to our previous studies.^37^, ^39^

### 2.6 The characterization of iPS‐MSC in accordance with the ISCT guidelines

Differentiations of the expanded iPS‐MSC into adipocytes, osteocytes and chondrocytes were examined (Figure S2). The human MSCs are characterized by the presence of the following positive and negative surface markers analysed by flow cytometric measurement or immunocytochemistry: ≥95% positive: CD105, CD73 and CD90 and ≤2% negative: CD45, CD34, CD14, CD19 and HLA‐DR (Figure S3).

### 2.7 In vitro study design for assessment of oxidative stress effect of p‐Cresol‐on inducing renal tubular epithelial cells damage

To elucidate the impact of oxidative stress on renal tubular epithelial cells (ie the NRK‐52E cells), these cells were co‐cultured with p‐Cresol (25 μmol/L, ie uraemic toxin producing oxidative stress to mimic the situation in advanced CKD). The cells (1 × 10^5^) per mL, which were utilized in this in vitro study, were first cultured in DMEM‐Low plus 10% FBS. For Western blotting, the cells were co‐cultured with designed drug in 10‐cm dish with 1.0 × 10^6^ cells for 24 hours. All the cells were then collected for individual assays. The dosage of p‐Cresol in the culture medium was based on our previous report.^40^

### 2.8 The time‐points of measurement of blood urine nitrogen (BUN) and creatinine levels

The blood samples were serially collected before and after the CKD procedure (ie at days 14 and 35 prior to and immediately before the animals to be killed). Serum levels of creatinine and BUN were measured in duplicate using standard laboratory equipment. The mean intra‐assay coefficient of variance for BUN and creatinine was less than 4.0%.

### 2.9 The time courses of collection of 24‐hour urine for the ratio of urine protein to urine creatinine

The procedure and protocol were based on our previous reports.^30^, ^32^, ^39^ For the collection of 24‐hour urine in individual study, each animal was put into a metabolic cage [DXL‐D, space: 190 × 290 × 550 mm^3^, Suzhou Fengshi Laboratory Animal Equipment Co. Ltd.] for 24 hours with free access to food and water. Urine in 24 hours was collected in all animals prior to and at days 14 and 35 after CKD induction for determining the ratio of urine protein to urine creatinine.

### 2.10 Western blot analysis of left kidney specimens

The procedure and protocol have been described in our previous reports.^30^, ^32^, ^39^ In details, primary antibodies against tumour necrosis factor (TNF)‐α (1:1000, Cell Signaling), phosphorylated nuclear factor (p‐NF)‐κB (1:1000, Abcam), tumour necrosis factor receptor–associated factor (TRAF) (1:2000, Abcam), toll‐like receptor (TLR)‐2 (1:1000, Abcam), TLR‐4 (1:1000, Novus), myeloid differentiation primary response 88 (MyD88) (1:1000, Abcam), inhibitory‐κB kinase beta (IKK‐ß) (1:1000, Cell Signaling), nuclear factor of kappa light polypeptide gene enhancer in B cells inhibitor alpha (IKBα) (1:1000, Cell Signaling), interleukin (IL)‐1ß (1:1000, Cell Signaling), IL‐6 (1:1000, Biorbyt), matrix metalloproteinase (MMP)‐9 (1:2000, Abcam), apoptosis signal–regulating kinase 1 (ASK1) (1:1000, Abcam), NOX‐1 (1:1500, Sigma‐Aldrich), NOX‐2 (1:1000, Sigma‐Aldrich), NOX4 (1:1000, Abcam), oxidized protein (1:150, Millipore), p22phox (1:1000, Abcam), cleaved caspase‐3 (c‐Csp3) (1:1000, Cell Signaling), mitochondrial Bax (mito‐Bax) (1:1000, Abcam), phosphorylated (p)‐ERKs (1:1000, Calbiochem), p‐JNK (1:1000, Abcam), p‐p38 (1:1000, Sigma) and ASK1 (1:1000, Abcam) were used. Signals were detected with horseradish peroxidase (HRP)–conjugated goat antimouse, goat anti‐rat, or goat anti‐rabbit IgG.

Immunoreactive bands were visualized by enhanced chemiluminescence (ECL; Amersham Biosciences), which was then exposed to Biomax L film (Kodak). For quantification, ECL signals were digitized using Labwork software (UVP). For oxyblot protein analysis, a standard control was loaded on each gel.

### 2.11 Immunofluorescent studies

The procedures and protocols for immunofluorescent (IF) examinations have been described in our previous reports.^30^, ^32^, ^39^ In details, IF staining was performed for the examinations of CD14 (1:200, Bioss antibodies) and CD68 (1:100, Abcam). Respective primary antibody was used with irrelevant antibodies as controls. Three sections of kidney specimens were analysed in each rat. For quantification, three randomly selected HPFs (200× for IHC and IF studies) were analysed in each section. The mean number per HPF for each animal was then determined by summation of all numbers divided by 9.

### 2.12 Histopathology scoring of kidney injury at day 35 after CKD induction

The histopathology scoring of kidney injury was determined in a blinded fashion as our previous reports.^30^, ^32^, ^39^ Briefly, the left kidney specimens from the animals in each group were fixed in 10% buffered formalin, embedded in paraffin, sectioned at 4 µm and stained (haematoxylin and eosin; H&E) for light microscopy. The score reflected the grading of tubular necrosis, loss of brush border, cast formation and tubular dilatation in 10 randomly chosen, non‐overlapping fields (200×) for each animal as follows: 0 (none), 1 (≤10%), 2 (11%‐25%), 3 (26%‐45%), 4 (46%‐75%) and 5 (≥76%).

### 2.13 Histopathological analysis for quantification of kidney fibrosis at day 35 after CKD induction

Masson's trichrome staining was utilized for investigating the fibrosis in kidney parenchyma. Three serial sections of kidney in each animal were prepared at 3 µm thickness by microtome (Leica RM2235). The integrated area (µm^2^) of fibrosis on each section was calculated using the Image Tool 3 (IT3) image analysis software (University of Texas, Health Science Center, San Antonio, UTHSCSA; Image Tool for Windows, Version 3.0). Three randomly selected high‐power fields (HPFs) (100×) were analysed in each section. The numbers of pixels in fibrotic area obtained from three HPFs were summated. The procedure was repeated in two other sections for each animal. The mean pixel number per HPF for each animal was then determined by summating all pixel numbers and dividing by 9. The mean integrated area (µm^2^) of fibrosis in kidney per HPF was obtained using a conversion factor of 19.24 (1 µm^2^ corresponded to 19.24 pixels).

### 2.14 Isolation of mitochondria for detection of mitochondrial Bax protein

The samples were excised and washed with buffer A (100 mmol/L Tris‐HCl, 70 mmol/L sucrose, 10 mmol/L EDTA and 210 mmol/L mannitol, pH 7.4). Samples were minced finely in cold buffer A and were incubated for 10 minutes. All samples were homogenized in an additional 3 mL of buffer A using a motor‐driven grinder. The homogenate was centrifuged twice at 700 *g* for 10 minutes at 4°C. The supernatant was centrifuged again at 8500 *g* for 15 minutes, and the pellets were then washed with buffer B (10 mmol/L Tris‐HCl, 70 mmol/L sucrose, 1 mmol/L EDTA and 230 mmol/L mannitol, pH 7.4). The mitochondria‐rich pellets were then collected and stored at −80°C for individual study.

### 2.15 Statistical analysis

Quantitative data are expressed as mean ± SD. Statistical analyses were performed using SAS statistical software for Windows version 8.2 (SAS Institute). Statistical differences between two groups were analysed with Student's *t* test was used. A probability value <.05 was considered statistically significant.

## 3 RESULTS

### 3.1 The protein expressions of inflammatory downstream signalling, oxidative stress and cell apoptosis/death signalling at 24/72 hours after oxidative stress stimulation in renal tubular epithelial cells

**FIGURE 1:**
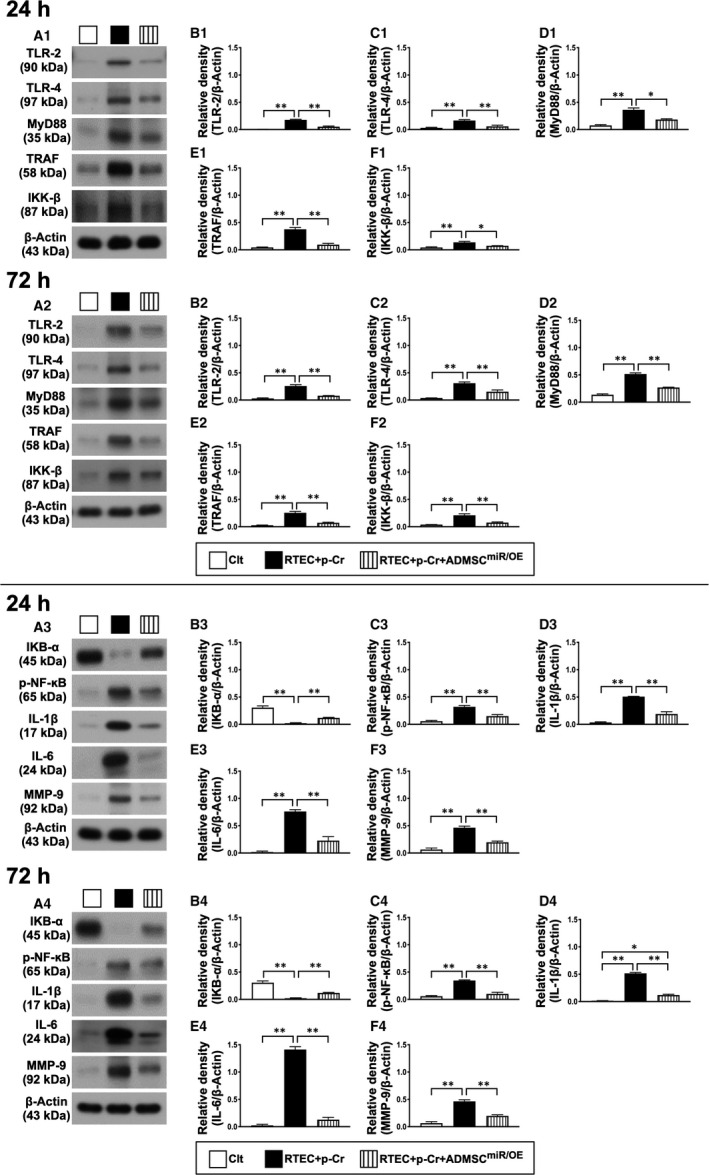
Impact of miR‐19a‐3p overexpression in iPS‐MSCs on protein expressions of inflammatory upstream signalling at 24 and 72 h after oxidative stress stimulation in NRK‐52E cells. A1‐F1, Western blot representative images (A1), protein expressions of toll‐like receptor (TLR)‐2 (B1), TRL4 (C1), myeloid differentiation primary response 88 (MyD88) (D1), tumour necrosis factor receptor–associated factor (TRAF) (E1) and Inhibitory‐κB kinase beta (IKK‐ß) (F1) at 24‐h cell culturing, *P < .01 and **P < .001. A2‐F2, Western blot representative images (A2), protein expressions of toll‐like receptor (TLR)‐2 (B2), TRL4 (C2), myeloid differentiation primary response 88 (MyD88) (D2), tumour necrosis factor receptor–associated factor (TRAF) (E2) and inhibitory‐κB kinase beta (IKK‐ß) (F2) at 48‐h cell culturing, *P < .01 and **P < .001. All statistical analyses were performed using Student's *t* test (n = 4 for each group). Clt = control group (ie NRK‐52E cells in culture medium); RTEC + p‐Cr = renal tubular epithelial cells (RTEC) (ie NRK‐52E cells) + p‐Cresol (25 μmol/L); and iPS‐MSC^miR/OE^ = miR‐19a‐3p overexpression in human inducible pluripotent stem cell–derived mesenchymal stem cell. A3‐F3, Western blot representative images (A3), protein expressions of nuclear factor of kappa light polypeptide gene enhancer in B cell inhibitor alpha (IKBα) (B3), phosphorylated nuclear factor (p‐NF)‐κB (C3), interleukin (IL)‐1ß (D3), IL‐6 (E3) and matrix metalloproteinase (MMP)‐9 (F3) at 24‐h cell culture, *P < .01 and **P < .001. A4‐F4, Western blot representative images (A4), protein expressions of nuclear factor of kappa light polypeptide gene enhancer in B cell inhibitor alpha (IKBα) (B4), phosphorylated nuclear factor (p‐NF)‐κB (C4), interleukin (IL)‐1ß (D4), IL‐6 (E4) and matrix metalloproteinase (MMP)‐9 (F4) at 48‐h cell culture, *P < .01 and **P < .01. Student's *t* test was used (n = 4 for each group). Clt = control group (ie NRK‐52E cells in culture medium); RTEC + p‐Cr = renal tubular epithelial cells (RTEC) (ie NRK‐52E cells) + p‐Cresol (25 μmol/L); and iPS‐MSC^miR/OE^ = miR‐19a‐3p overexpression in human inducible pluripotent stem cell–derived mesenchymal stem cell

In the in vitro study, NRK‐52E cells were co‐cultured with p‐Cresol (25 μmol/L, ie an indicator of oxidative stress) for elucidating whether miR‐19a‐3p‐transfected iPS‐MSCs would suppress the spreading of the inflammatory, oxidative stress and cell apoptosis/death signalling. As we expected, the protein expressions of TLR2, TRL4, MyD88, TRAF6, IKK‐ß, p‐NFκB, IL‐1ß, IL‐6 and MMP‐9, nine biomarkers of downstream inflammatory signalling, were significantly increased in NRK‐52E cells treated by p‐Cresol (ie group 2) than in control group (ie group 1, only NRK‐52E cells in culture medium) and were significantly reversed in NRK‐52E cells treated by p‐Cresol (25 μmol/L) + miR‐19a‐3p‐transfected iPS‐MSCs (ie group 3). On the other hand, the protein expression of IKBα, a protein for transmitting the inflammatory signalling from the upper to the lower pathway, exhibited an opposite pattern of TLR4/MyD88 among the three groups, implying a utilization of p‐IKBα was notably required with respect to inflammatory reaction that was ameliorated by MSC therapy (Figure 1).

**FIGURE 2:**
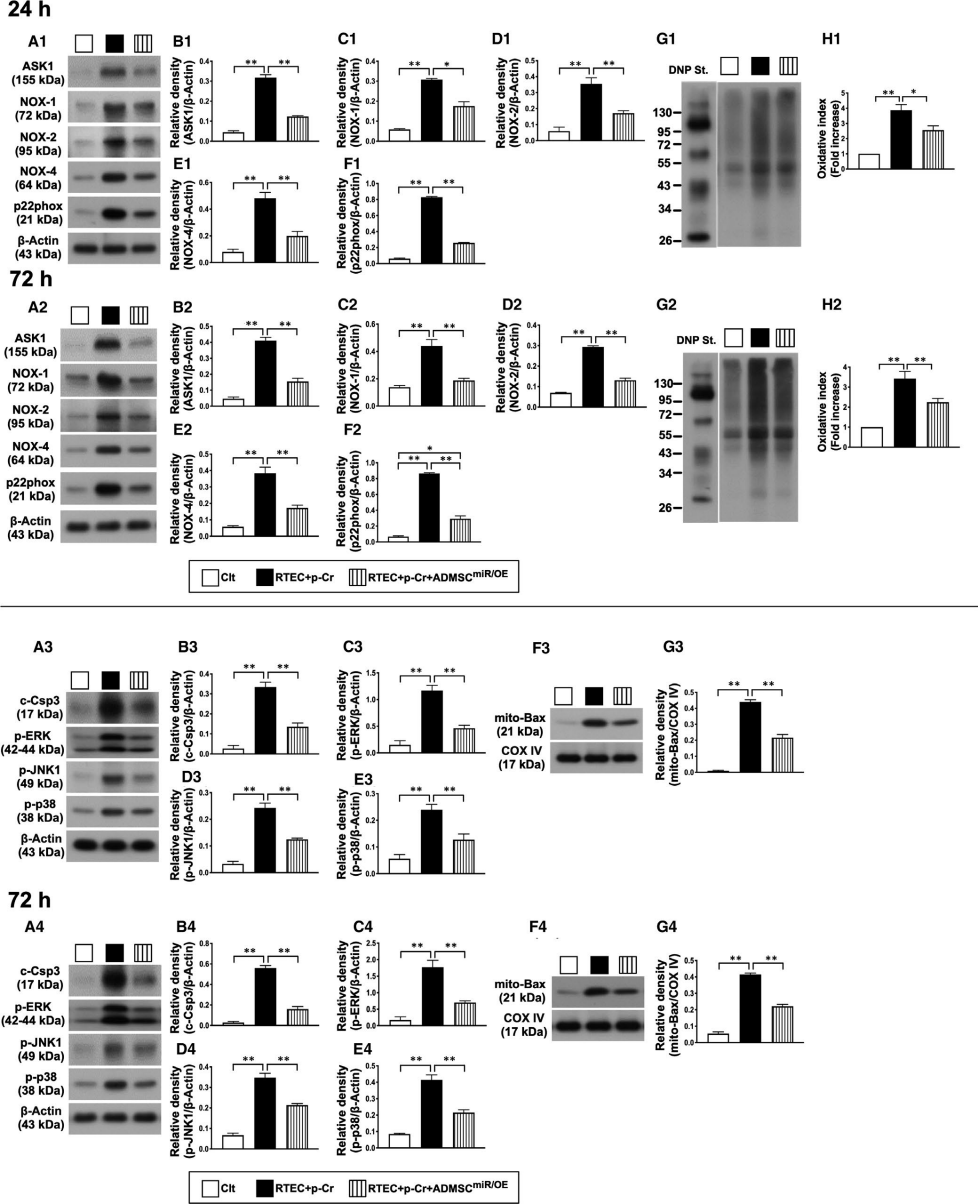
Impact of miR‐19a‐3p overexpression in iPS‐MSCs on protein expressions of oxidative stress at 24 and 72 h after oxidative stress stimulation in NRK‐52E cells. A1‐H1, Western blot representative images (A1), protein expressions of apoptosis signal–regulating kinase 1 (ASK1) (B1), NOX‐1 (C1), NOX‐2 (D1), NOX4 (E1), oxidized protein (G1‐H1) and p22phox (F1) at 24‐h cell culture, *P < .01 and **P < .001. A2‐F2, Western blot representative images (A2), protein expressions of apoptosis signal–regulating kinase 1 (ASK1) (B2), NOX‐1 (B3), NOX‐2 (D2), NOX4 (E2), oxidized protein (G2‐H2) and p22phox (F2) at 48‐h cell culture, *P < .01 and **P < .001. Clt = control group (ie NRK‐52E cells in culture medium); RTEC + p‐Cr =renal tubular epithelial cells (RTEC) (ie NRK‐52E cells) + p‐Cresol (25 μmol/L); and iPS‐MSC^miR/OE^ = miR‐19a‐3p overexpression in human inducible pluripotent stem cell–derived mesenchymal stem cell. A3‐G3, Western blot representative images (A3), protein expressions of cleaved caspase‐3 (c‐Csp3) (B3), mitochondrial Bax (mito‐Bax) (F3‐G3), phosphorylated (p)‐ERKs (C3), p‐JNK (D3) and p‐p38 (E3) at 24‐h cell culture, *P < .01 and **P < .001. A4‐G4, Western blot representative images (A4), protein expressions of cleaved caspase‐3 (c‐Csp3) (B4), mitochondrial Bax (mito‐Bax) (F4‐G4), phosphorylated (p)‐ERKs (C4), p‐JNK (D4) and p‐p38 (E4) at 72‐h cell culture, *P < .01 and **P < .001. Student's *t* test was used (n = 4 for each group). Clt = control group (ie NRK‐52E cells in culture medium); RTEC + p‐Cr = renal tubular epithelial cells (RTEC) (ie NRK‐52E cells) + p‐Cresol (25 μmol/L); and iPS‐MSC^miR/OE^ = miR‐19a‐3p overexpression in human inducible pluripotent stem cell–derived mesenchymal stem cell

In the aforementioned condition, we performed the Western blot analysis and the results demonstrated that the protein expressions of ASK1, NOX‐1, NOX‐2, NOX4, oxidized protein and p22phox, six indices of oxidative stress, displayed an identical pattern of TLR‐4/MyD88 among the three groups (Figure 2).

To further assess the cell apoptosis/death biomarkers in the above‐mentioned situation, Western blot analysis was once more utilized and the results consistently displayed that the protein expressions of cleaved caspase‐3, mitochondrial Bax, p‐ERKs, p‐JNK and p‐p38 exhibited a similar pattern of TLR‐4/MyD88 among the three groups (Figure 2).

**FIGURE 3:**
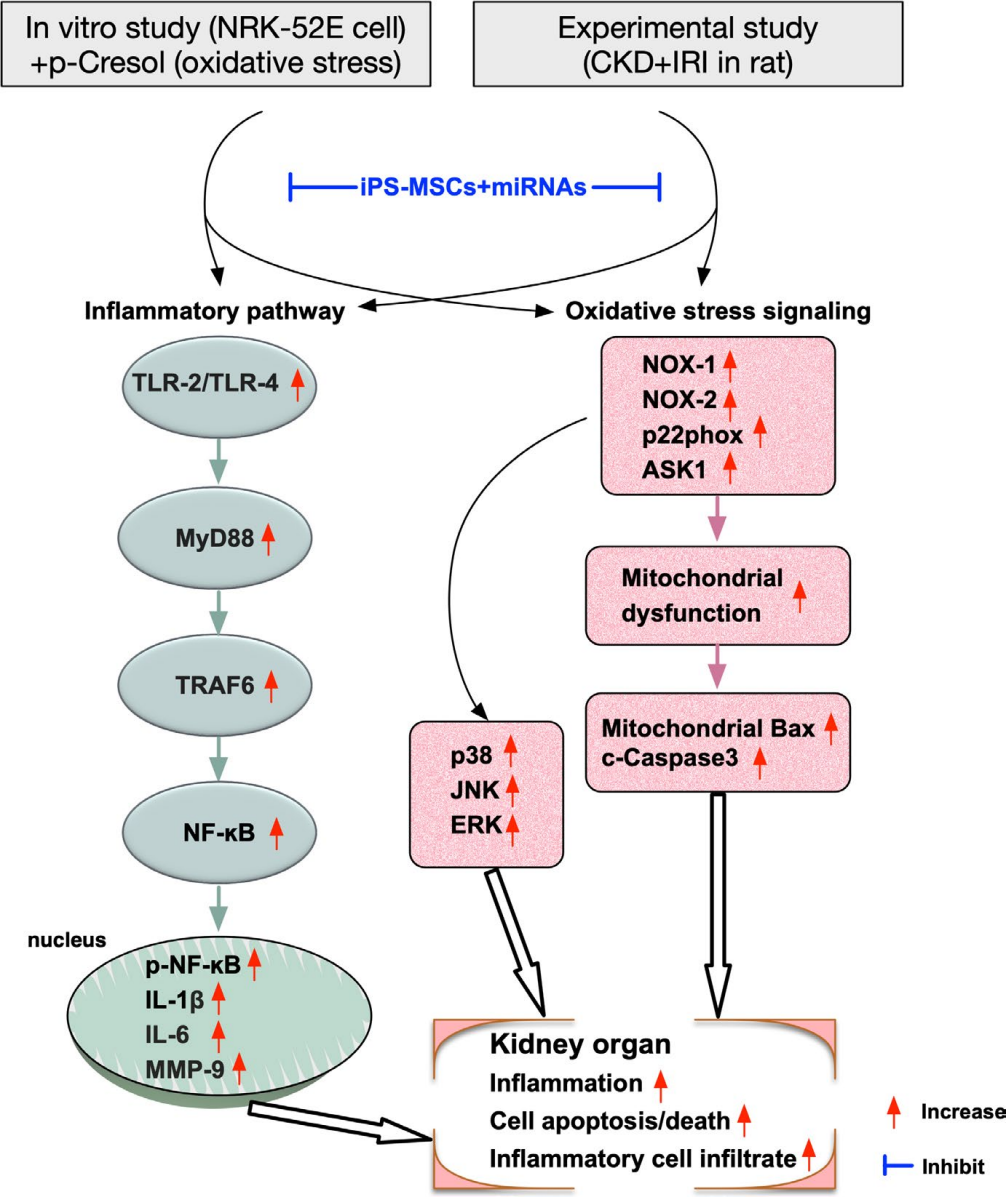
The proposed mechanism of miR‐19a‐3p overexpression in iPS‐MSCs on protecting the renal tubular epithelial cells against oxidative stress damage via down‐regulating the inflammatory‐oxidative stress and cell apoptotic/death signalling pathways through summarizing the results from Figures 1, 2

Based on the findings from Figures 1, 2, we proposed the underlying mechanism of miR‐overexpression in iPS‐MSCs on protecting the renal tubular epithelial cells through regulating the inflammatory‐oxidative stress‐cell apoptosis/death signalling pathways in Figure 3.

### 3.2 The time courses of circulating levels of creatinine and blood urine nitrogen (BUN) and ratio of urine protein to urine creatinine prior to and after CKD‐IR procedures

**FIGURE 4:**
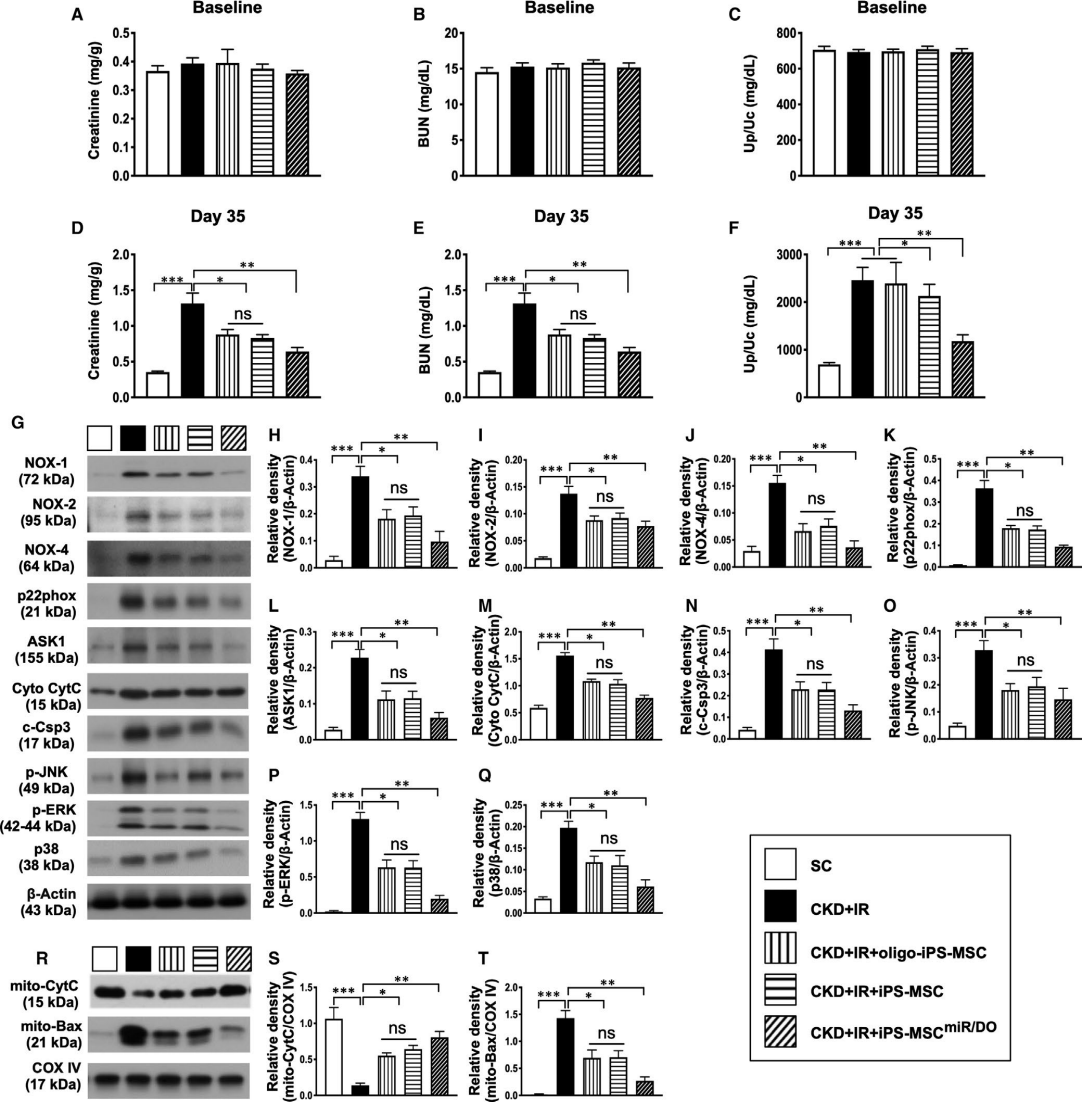
Plasma levels of creatinine and blood urine nitrogen (BUN), ratio of urine protein to urine creatinine, protein expressions of oxidative stress and apoptosis/cell death biomarkers at baseline and at day 35 after CKD‐IR procedure. A, Creatinine level at day 0, *P* > .5. B, BUN level at day 0, *P* > .5. C, Ratio of urine protein (Up) to urine creatinine (Uc) at day 0, *P* > .5. D, Creatinine level at day 35, *P < .01; **P < .001; and ***P < .0001. E, BUN level at day 35, *P < .01; **P < .001; and ***P < .0001. F, Ratio of Up to Uc at day 35, *P < .01; **P < .001; and ***P < .0001. G, Western blot representative images. H, Protein expression of NOX‐1, *P < .01; **P < .001; and ***P < .0001. I, Protein expression of NOX‐2, *P < .01; **P < .001; and ***P < .0001. J, Protein expression of NOX‐4, *P < .01; **P < .001; and ***P < .0001. K, Protein expressions of p22phox, *P < .01; **P < .001; and ***P < .0001. L, Protein expression of ASK1, *P < .01; **P < .001; and ***P <.0001. M, Protein expression of cytosolic cytochrome C (cyt‐Cyto‐C), *P < .01; **P < .001; and ***P < .0001. N, Protein expression of cleaved caspase‐3 (c‐Csp3), *P < .01; **P < .001; and ***P < .0001. O, Protein expression of p‐JNK, *P < .01; **P < .001; and ***P < .0001. P, Protein expression of p‐EKR, *P < .01; **P < .001; and ***P < .0001. Q, Protein expression of p‐p38, *P < .01; **P < .001; and ***P < .0001. R, Western blot representative images. S, Protein expression of mitochondrial cytochrome C (mit‐Cyto‐C), *P < .01; **P < .001; and ***P < .0001. T, Protein expression of mitochondrial (mito)‐Bax, *P < .01; **P < .001; and ***P < .0001. Student's *t* test was used (n = 6 for each group). ns = no significant difference. SC = sham‐operated control; CDK‐IR = chronic kidney disease + ischaemia‐reperfusion; iPS‐MSC = human inducible pluripotent stem cell–derived mesenchymal stem cell; and MSC^miR/DOE^ = miR‐19a‐3p and miR‐20a‐5p overexpression (ie double miRNA overexpression) in iPS‐MSCs

By day 0, the circulating levels of creatinine, BUN and the ratio of urine protein to urine creatinine did not differ among the five groups (Figure 4A‐C). However, by day 14 after CKD induction, these parameters were significantly lower in group 1 (SC) than in group 2 (CKD + IR), group 3 (CKD + IR + oligo‐miR^DOE^ of iPS‐MSCs), group 4 (CKD + IR + iPS‐MSCs) and group 5 (CKD + IR + miR^DOE^ of iPS‐MSCs). On the other hand, these parameters did not differ among groups 2 to 5.

By day 35 after CKD induction, theses parameters were lowest in group 1, highest in group 2, significantly lower in group 5 than in groups 3 and 4, but they did not differ between groups 3 and 4 (Figure 4D‐F).

### 3.3 The protein expressions of oxidative stress and mitochondrial‐damaged biomarkers in renal parenchyma at day 35 after CKD‐IR procedure

To further assess the change of oxidative stress signalling in situation of CKD‐IR and the impact of miR^DOE^ + iPS‐MSC treatment on protecting the kidney against the CKD‐IR, the Western blot was performed again. The results showed that the protein expressions of NOX‐1, NOX2, NOX‐4, p22phox and ASK1, five indices of oxidative stress, were lowest in group 1, highest in group 2, significantly lower in group 5 than in groups 3 and 4, but they did not differ between groups 3 and 4 (Figure 4G‐L).

Additionally, the protein expression of cytochrome C was analysed for determining the mitochondrial function. As our expected, the protein expression of cytosolic cytochrome C, an indicator of mitochondrial damage, exhibited a similar pattern, whereas the protein expression of mitochondrial cytochrome C, an indicator of mitochondrial functional integrity, exhibited an opposite pattern of oxidative stress among the groups (Figure 4M, S).

### 3.4 The protein expressions of apoptosis/cell death biomarkers in renal parenchyma at day 35 after CKD‐IR procedure

The protein expressions of cleaved caspase‐3, mitochondrial Bax (ie apoptotic markers), p‐JNK, p‐EKR and p‐38 (ie cell proliferation/death biomarkers) were lowest in group 1, highest in group 2, significantly lower in group 5 than in groups 3 and 4, but they did not differ between groups 3 and 4 (Figure 4N‐Q, T).

### 3.5 The protein expressions of inflammatory biomarkers in renal parenchyma at day 35 after CKD‐IR procedure

**FIGURE 5:**
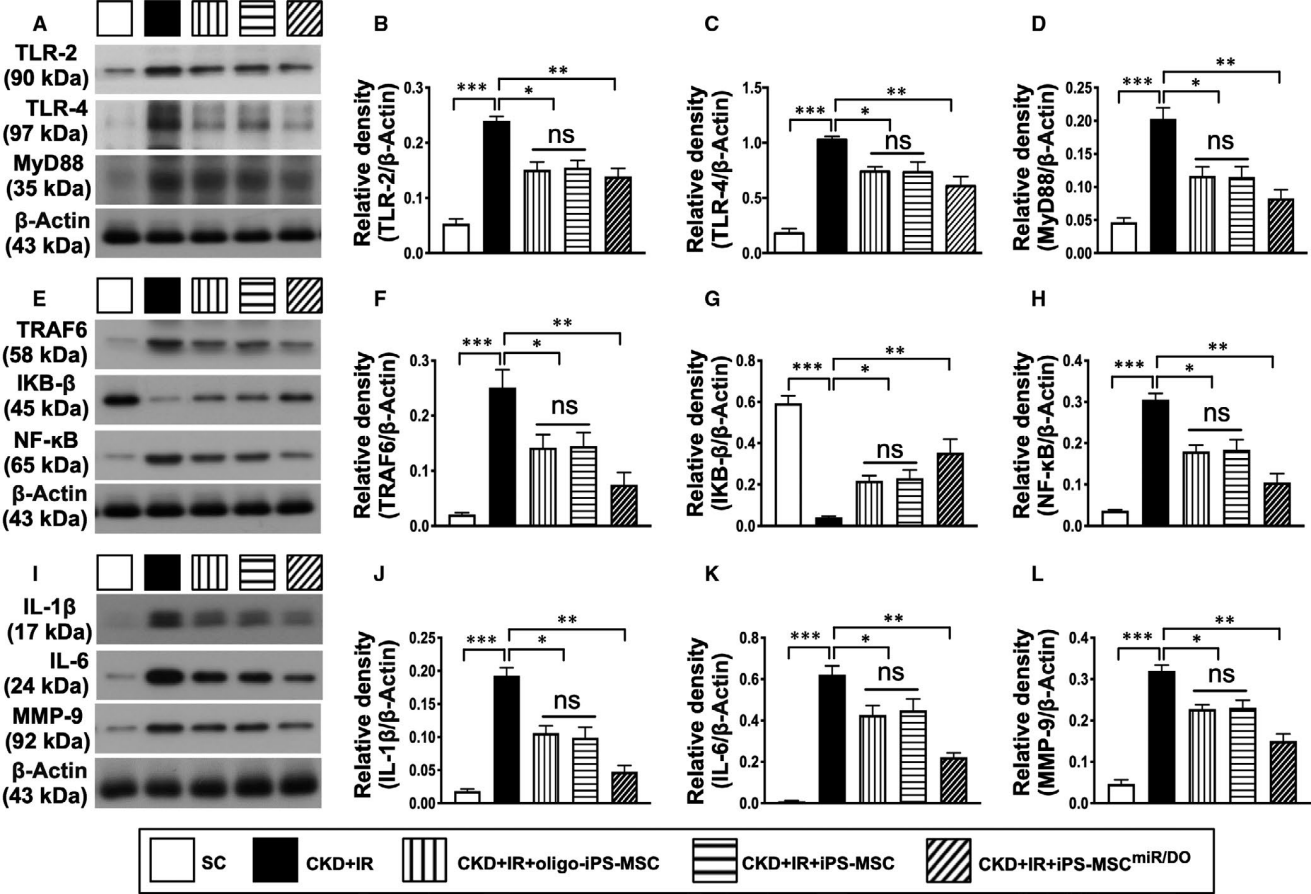
The protein expressions of inflammatory biomarkers in kidney parenchyma at day 35 after CKD‐IR procedure. A, Western blot representative images. B, Protein expression of TLR‐2, *P < .01; **P < .001; and ***P < .0001. C, Protein expression of TLR‐4, *P < .01; **P < .001; and ***P < .0001. D, Protein expression of MyD88, *P < .01; **P < .001; and ***P < .0001. E, Western blot representative images. F, Protein expression of TRAF6, *P < .01; **P < .001; and ***P < .0001. G, Protein expression of IKB‐ß, *P < .01; **P < .001; and ***P < .0001. H, Protein expression of phosphorylated (p)‐NF‐κB, *P < .01; **P < .001; and ***P < .0001. I, Western blot representative images. J, Protein expression of IL‐1ß, *P < .01; **P < .001; and ***P < .0001. K, Protein expression of IL‐6, *P <.01; **P <.001; and ***P <.0001. L, Protein expression of MMP‐9, *P <.01; **P < .001; and ***P < .0001. Student's *t* test was used (n = 6 for each group). ns = no significant difference. SC = sham‐operated control; CDK‐IR = chronic kidney disease + ischaemia‐reperfusion; iPS‐MSC = human inducible pluripotent stem cell–derived mesenchymal stem cell; and MSC^miR/DOE^ = miR‐19a‐3p and miR‐20a‐5p overexpression (ie double miRNA overexpression) in iPS‐MSCs

To elucidate whether expression of inflammatory signalling was similar in vivo to that of the in vitro, Western blot analysis was performed for the harvested kidney specimen. The protein expressions of TLR‐2, TLR‐4, MyD88, TRAF6, p‐NF‐κB, IL‐1ß, IL‐6 and MMP‐9, eight indicators of inflammatory biomarkers, were lowest in group 1, highest in group 2, significantly lower in group 5 than in groups 3 and 4, but they did not differ between groups 3 and 4. On the other hand, the protein expression of IKB‐ß, a protein for propagating the inflammatory signalling from the upper to the lower pathway, revealed an opposite pattern of TLR‐4/MyD88 among the five groups (Figure 5).

### 3.6 The kidney injury score and fibrotic area in renal parenchyma at day 35 after CKD‐IR procedure

**FIGURE 6:**
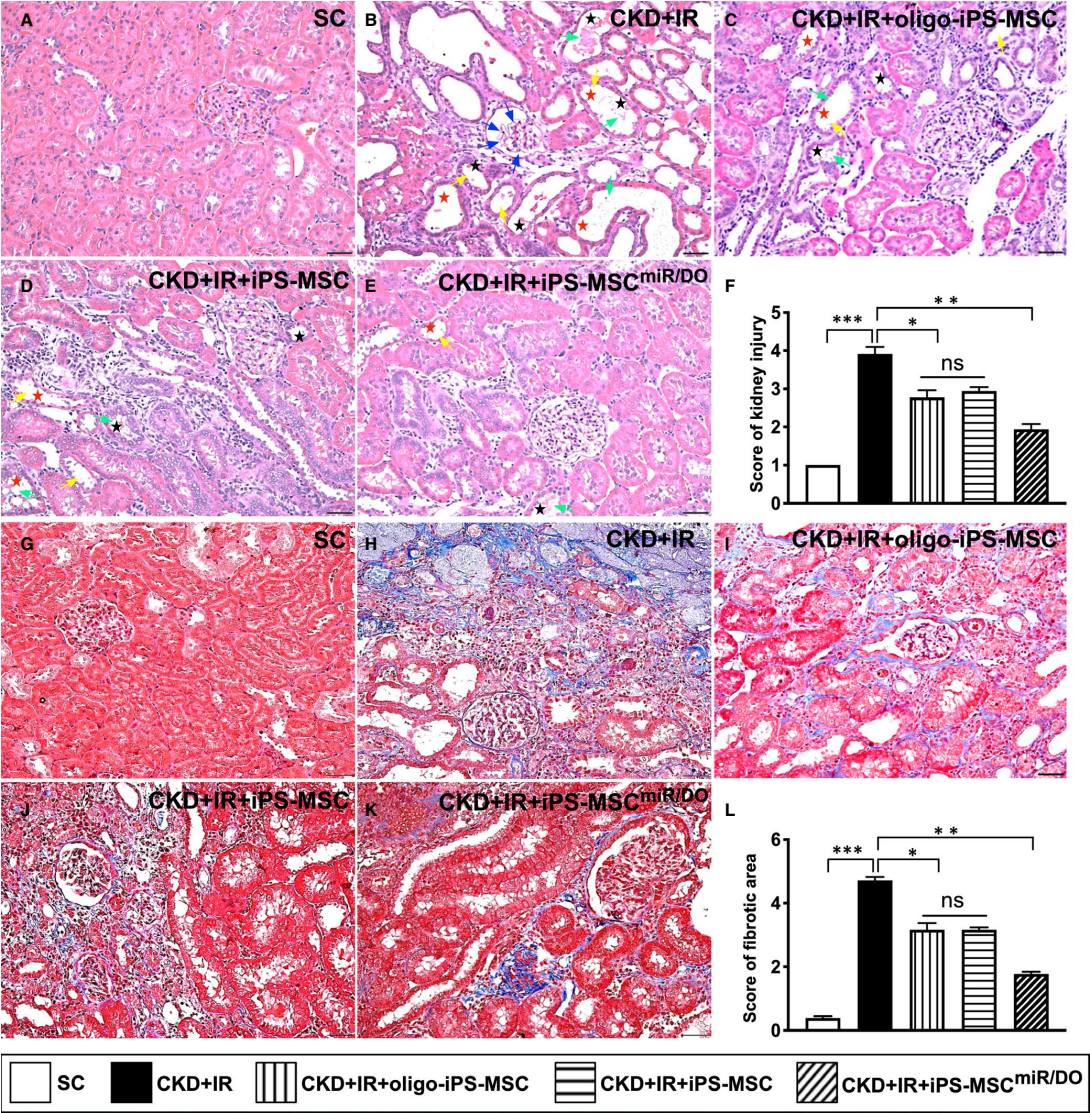
The kidney injury score and fibrotic area in kidney parenchyma at day 35 after CKD‐IR procedure. A‐E, Light microscopic findings (200×) of HE stain showing significantly higher degree of loss of brush border in renal tubules (yellow arrows), tubular necrosis (green arrows), tubular dilatation (red asterisk) protein cast formation (black asterisk) and dilatation of Bowman's capsule (blue arrows) in CKD‐IR group than in other groups. F, Analytical result of kidney injury score, *P < .01; **P < .001; and ***P < .0001. G‐K, Illustrating histological finding (200×) of Masson's trichrome stain for fibrotic area of kidney parenchyma (blue colour). L, Analytical result of fibrotic area, *P < .01; **P < .001; and ***P < .0001. Scale bars in right lower corner represent 50 µm. Student's *t* test was used (n = 6 for each group). ns = no significant difference. SC = sham‐operated control; CDK‐IR = chronic kidney disease + ischaemia‐reperfusion; iPS‐MSC = human inducible pluripotent stem cell–derived mesenchymal stem cell; and MSC^miR/DO^ = miR‐19a‐3p and miR‐20a‐5p overexpression (ie double miRNA overexpression) in iPS‐MSCs

The microscopic finding of HE stain demonstrated that the kidney injury score was lowest in group 1, highest in group 2, significantly lower in group 5 than in groups 3 and 4, but it did not differ between groups 3 and 4. Additionally, the Masson's trichrome stain identified that the expression of fibrotic area was identical to the pattern of kidney injury score among the five groups (Figure 6).

### 3.7 The inflammatory cell infiltration in renal parenchyma at day 35 after CKD‐IR procedure

**FIGURE 7:**
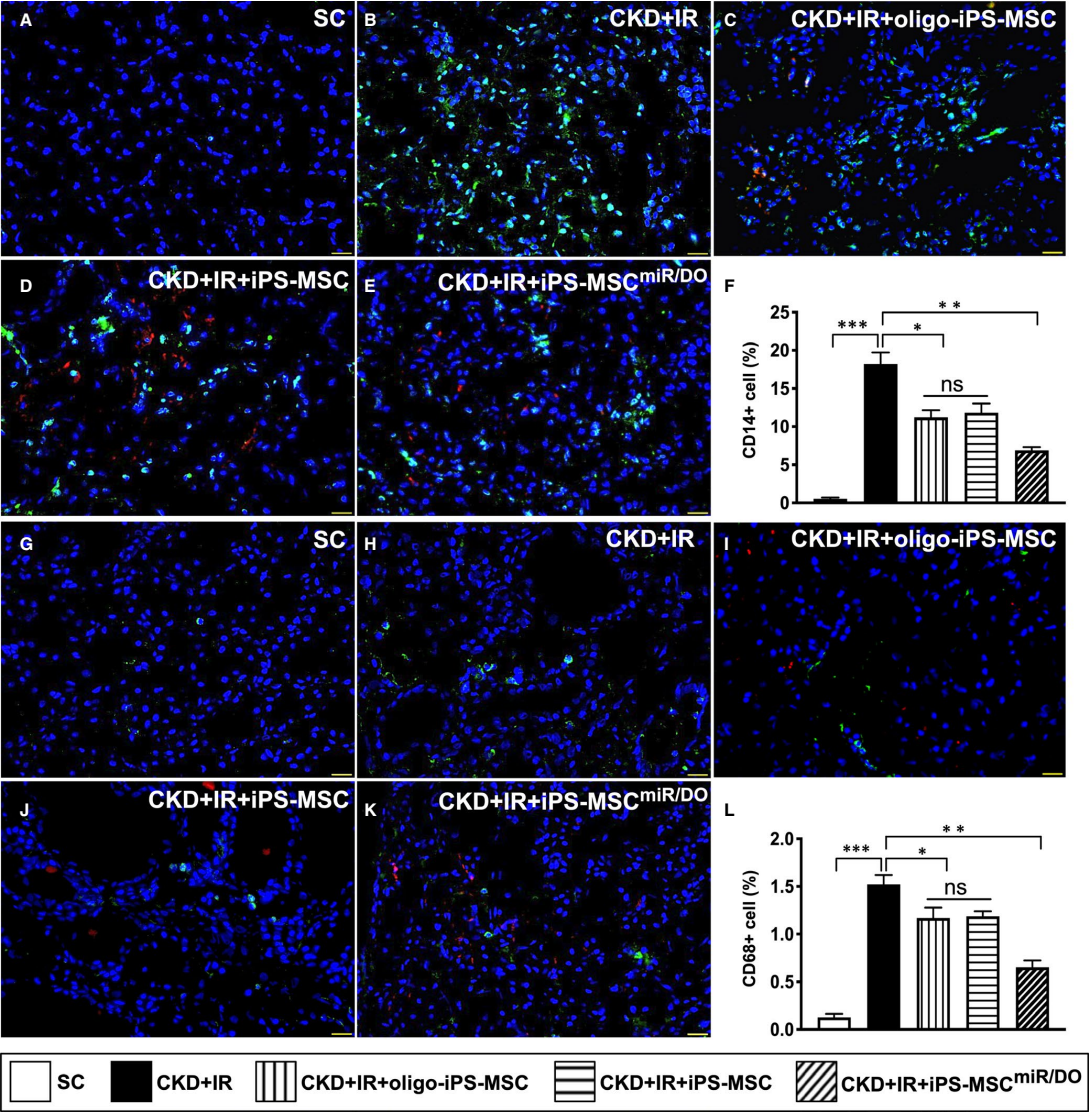
The inflammatory cell infiltration in kidney parenchyma at day 35 after CKD‐IR procedure. A‐E, Illustrating the immunofluorescent (IF) microscopic finding for identification of CD14+ cells (green colour). F, Analytical result of number of CD14+ cells, *P < .01; **P < .001; and ***P < .0001. G‐K, Illustrating the IF microscopic finding for identification of CD68+ cells (green colour). L, Analytical result of number of CD68+ cells, *P < .01; **P < .001; and ***P < .0001. Student's *t* test was used (n = 6 for each group). ns = no significant difference. SC = sham‐operated control; CDK‐IR = chronic kidney disease + ischaemia‐reperfusion; iPS‐MSC = human inducible pluripotent stem cell–derived mesenchymal stem cell; and MSC^miR/DOE^ = miR‐19a‐3p and miR‐20a‐5p overexpression (ie double miRNA overexpression) in iPS‐MSCs

The protein expressions of CD14+ and CD68+ in kidney parenchyma, two indications of inflammation, were lowest in group 1, highest in group 2, significantly lower in group 5 than in groups 3 and 4, but they did not differ between groups 3 and 4 (Figure 7).

## 4 DISCUSSION

This study which investigated (i) the impact of miR‐19a‐3p‐transfected iPS‐MSCs on protecting the NRK‐52E cells against uraemic substance damage and (ii) whether miR^DOE^ of iPS‐MSC therapy would be superior to iPS‐MSC alone for preserving residual renal function in CKD rat suffering acute kidney IR injury yielded several striking implications. First, in vitro results demonstrated that miR‐19a‐3p overexpression of iPS‐MSCs significantly reduced the p‐Cresol damaged NRK‐52E cells mainly through the down‐regulating the inflammatory and oxidative stress signallings. Second, in vivo study demonstrated that inflammatory and oxidative stress downstream signallings were substantially up‐regulated in CKD followed by IR injury. However, these signalling pathways were significantly suppressed by iPS‐MSCs and further significantly suppressed by miR^DOE^ of iPS‐MSCs. Third, of importance was that miR^DOE^ of iPS‐MSCs was superior to iPS‐MSCs only on preserving the residual renal function in the setting of CKD concomitant with acute IR injury.

Vast data have reported that inflammatory reaction and oxidative stress are the two extremely important contributors of endothelial dysfunction, arterial atherosclerosis and obstruction and ischaemia‐relayed organ dysfunction.^39^, ^41^, ^42^, ^43^ Additionally, plentiful experimental studies have further demonstrated that these two parameters directly participate in ischaemia‐related and IR‐related organ damage, resulting in organ dysfunction.^7^, ^30^, ^32^, ^39^ As we expected in the present in vivo study, the acute kidney IR procedure actually worsened the residual renal function in pre‐existence of CKD. One important finding in the present study was that CKD + IR substantially enhanced the oxidative stress and inflammatory downstream signallings, resulting in increases in kidney injury score and fibrosis in kidney parenchyma (ie histopathological findings) as well as the apoptosis and mitochondrial damage in protein level of kidney specimen. These findings, in addition to corroborating the findings of previous studies,^7^, ^30^, ^32^, ^39^ could explain why the circulatory BUN and creatinine and the ratio of urine protein to urine creatinine (ie indices of renal functional integrity) remarkably increased in CKD + IR animals than in those of SC animals.

Interestingly, our previous studies have shown that ADMSCs^7^, ^30^, ^37^ and iPS‐MSC^38^ therapies effectively protected kidney architecture and function against IR injury in rodent. Additionally, our recent study has revealed that iPS‐MSC therapy also effectively preserved the renal function in CKD setting.^39^ In this experimental study, we also found that iPS‐MSC therapy significantly protected the renal ultrastructural integrity and residual renal function in CKD + IR animals. In this way, our results were comparable with those of our previous and recent studies,^7^, ^30^, ^37^, ^39^ suggesting that both ADMSCs and iPS‐MSCs may have comparable capacity to protect the organ from IR injury.

Inflammation and oxidative stress are the two fundamental signalling pathways that commonly participate in mitochondrial dysfunction and cell apoptosis and death resulting in organ dysfunction, fibrosis and failure.^7^, ^30^, ^32^, ^37^, ^39^ Additionally, abundant data have clearly identified that inflammation and oxidative stress play two crucial roles on eliciting and propagating the renal parenchymal disease, tubular interstitial fibrosis and glomerulus sclerosis, resulting in CKD and progressively renal function deterioration.^30^, ^32^, ^39^ Based on our in vitro study, we found that p‐Cresol (ie a uraemic toxic substance) treatment markedly up‐regulated the protein expressions of inflammatory and oxidative stress biomarkers in NRK‐52E cells. Additionally, the cellular apoptosis and cell death signalling were found to be increased in p‐Cresol‐treated NRK‐52E cells. Our findings, therefore, were consistent with those of previous studies.^30^, ^32^, ^39^ An essential finding in the present study was that iPS‐MSCs significantly suppressed p‐Cresol‐induced inflammatory and oxidative stress downstream signallings. Our findings act in concert with those of previous studies which have demonstrated that MSCs, including ADMSC and iPS‐MSCs, have comparable capacity of immunomodulation and anti‐inflammation as well as tissue regeneration.

It is a universal concept that ischaemic zone frequently with limited blood flow and nutrient always elicits persistent oxidative stress and inflammation and generates free radicals unfavourable for the stem cell differentiation, proliferation and survival. Our previous study has shown that the microRNA expressions of miR‐19a‐3p and miR‐20a‐5p retained anti‐apoptotic property.^33^ Intriguingly, a cardinal finding in the present study was that the double overexpression of these two microRNAs was superior to iPS‐MSCs alone for preserving the residual kidney function and integrity of kidney architecture in CKD + IR in rat through down‐regulating the inflammatory, oxidative stress and apoptotic signalling pathways. In this way, our finding, in addition to extending that of our previous study,^33^ further suggested that this could be attributed to the anti‐apoptotic effect that enhanced the survival rate and functional integrity of iPS‐MSCs, especially in ischaemic situation.

It is well recognized that the residual renal function is a crucial factor for predicting the long‐term outcome in CKD patients. Regrettably, the residual renal function in those CKD patients is more vulnerable to be damaged by various harmful factors (ie called AKI in setting of CKD) which frequently results in poorer prognostic outcome than in those AKI patients with a pre‐existing normal renal function. Importantly, there is still lacking an effective treatment for these patients which, as a consequence, leads the majority of the patients are waiting for haemodialysis. In this way, our findings may be the potentially therapeutic candidate for these patients, especially those are refractory to the conventional therapy.

### 4.1 Study limitations

This study has limitation. First, the duration of IR study period in pre‐existing CKD setting was only 7 days. Thus, the long‐term outcome after IR injury was not investigated in the present study. Second, although extensive works had been done in the present study, the crystal‐clear mechanism of how iPS‐MSC and miR^DOE^ of iPS‐MSCs safeguarded the residual renal function and the integrity of kidney parenchyma remained uncertain. Based on the results of our study, we had schematically proposed the underlying mechanisms on how the miR^DOE^ of iPS‐MSCs affects and preserves the outcome in rodent in setting of CKD + IR injury in Figure 5. Third, this study did not measure the time courses of the parameters which were related to the serial changes of renal function in blood and urine after IR procedure. However, the final results (ie by day 7 after IR procedure) were still attractive and promising, suggesting that our study is still clinically relevant.

## 5 CONCLUSIONS

In conclusion, the miR^DOE^ of iPS‐MSCs provided remarkably additional benefits than iPS‐MSCs alone for recovering the residual kidney function and integrity of kidney architecture in CKD‐IR in rat through down‐regulating the inflammatory, oxidative stress and cell apoptotic/death signalling pathways as well as safeguarding the mitochondrial function.

## CONFLICTS OF INTEREST

All authors have read the journal's policy on disclosure of potential conflicts of interest and the journal's authorship agreement. The authors declare that they have no conflicts of interest. The article has been reviewed by and approved by all named authors.

## AUTHOR CONTRIBUTION

**Mel S. Lee:** Conceptualization (equal); Data curation (equal). **Hon‐Kan Yip:** Writing‐original draft (equal); Writing‐review & editing (equal). **Chih‐Chao Yang:** Resources (equal); Supervision (equal); Visualization (equal). **John Y. Chiang:** Writing‐original draft (equal); Writing‐review & editing (equal). **Tien‐Hung Huang:** Investigation (equal); Methodology (equal); Validation (equal); Visualization (equal). **Yi‐Chen Li:** Investigation (equal); Methodology (equal); Software (equal); Supervision (equal). **Kuan‐Hung Chen:** Methodology (equal); Writing‐original draft (equal). **Pei‐Hsun Sung:** Conceptualization (equal); Data curation (equal); Supervision (equal); Validation (equal); Writing‐original draft (equal).

## ETHICAL APPROVAL AND CONSENT TO PARTICIPATE

All animal procedures were approved by the Institute of Animal Care and Use Committee at Kaohsiung Chang Gung Memorial Hospital (Affidavit of Approval of Animal Use Protocol No. 2017102401) and performed in accordance with the Guide for the Care and Use of Laboratory Animals, 8th edition (NIH publication No. 85‐23, National Academy Press, Washington, DC, USA, revised 2011).

## Supporting information

Supplementary Material

## Data Availability

The data that support the findings of this study are available from the corresponding authors upon reasonable request.
