# Supplementary material for: Overexpression of miR‐19a and miR‐20a in iPS‐MSCs preserves renal function of chronic kidney disease with acute ischaemia‐reperfusion injury in rat
Source: J Cell Mol Med. 2021 Jun 23;25(16):7675–89. doi: 10.1111/jcmm.16613 (PMC8358869; doi:10.1111/jcmm.16613)

**Supplementary Results**

**Overexpression of miR-19a and miR-20a in iPS-MSC**

Single transfection and co-transfection with 25 nM miR-19a (Qiagen, miScript miRNA Mimics Syn-has-19a-3p, Cat. MSY0000073) and 25 nM miR-20a (Qiagen, miScript miRNA Mimics Syn-has-20a-5p, Cat. MSY0000075) mimics were performed in iPS-MSC through TransIT-X2 Dynamic Delivery System (Mirus, Cat. MIR6000) according to the manufacturer’s instructions. One day after transfections, cells were collected and assayed for individual miRNA expression through RT-qPCR. Total RNA was extracted using the miRNeasy mini kit (Qiagen Cat. 217004) in accordance to the protocol of the manufacturer. For mature miRNA quantification, reverse transcription was performed by miScript II RT kit (Qiagen, Cat. 218161). The expression of mature miRNAs was quantified by miScript SYBR Green PCR assay (Qiagen, Cat. 218073) and normalized by small nucleolar RNA, RNU6. Triplicate assays for each sample were performed on Step One-Plus machine (ABI).

**Supplementary Figure 1:**  Left panel showed that the level of mature miR-19a determined by RT-qPCR in each group, including control (scramble), miR-19a (single miR-19a mimic transfection), miR-20a (single miR-20a mimic transfection), miR-19a+ miR-20a (co-transfection of miR-19a and miR-20a), respectively. Similarly, the right panel showed that the level of mature miR-20a determined by RT-qPCR in the same grouping. We observed that single transfection of miR-19a did not interfere with the expression of miR-20a and vice versa. The co-transfection of miR-19a and miR-20a mimics resulted in a dramatic increase of the corresponding miRNA in iPS-MSC compared to control. Additionally, transfection with miR-19a and/or miR-20a mimics did not affect the viability of iPS-MSC in normal conditions.

**Supplemental Figure 2. Illustrating the results of iPSC derived MSCs differentiated into adipocytes, chondrocytes and osteocytes**

**A to C)** Microscopic findings (40x, 100x, 200x) of positively oil-red O stained adipocytes.  **D to F)** Microscopic findings (40x, 100x, 200x) for positively Alcian-Blue stained chondrocytes. **G to I)** Microscopic findings (40x, 100x, 200x) of positively Alizarin-Red S stained osteocytes.

**Supplementary Figure 3. Illustrating the flow cytometric graphs and plots for verifying the purity and complete differentiation of iPSC to MSC**

A1 to A3 illustrating the flow cytometric result of three typical iPSC surface markers. On the other hand, B1 to B4 demonstrating the flow cytometric result of four typical iPSC-MSCs surface markers.

**Supplementary Figure 1**

**
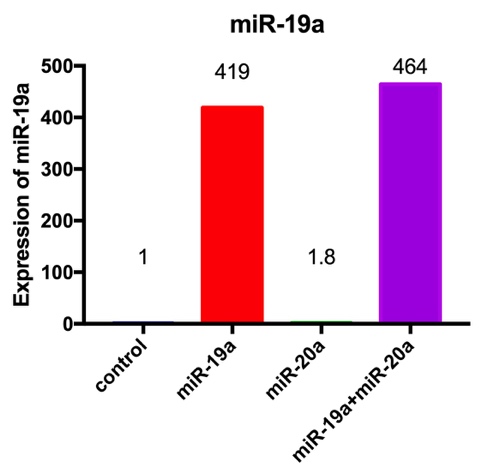

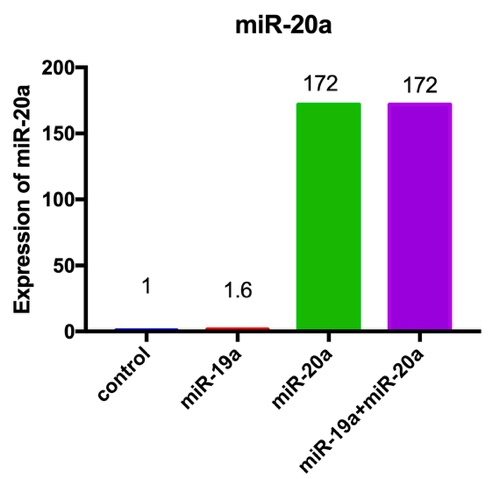
**

**Supplementary Figure 2**

**
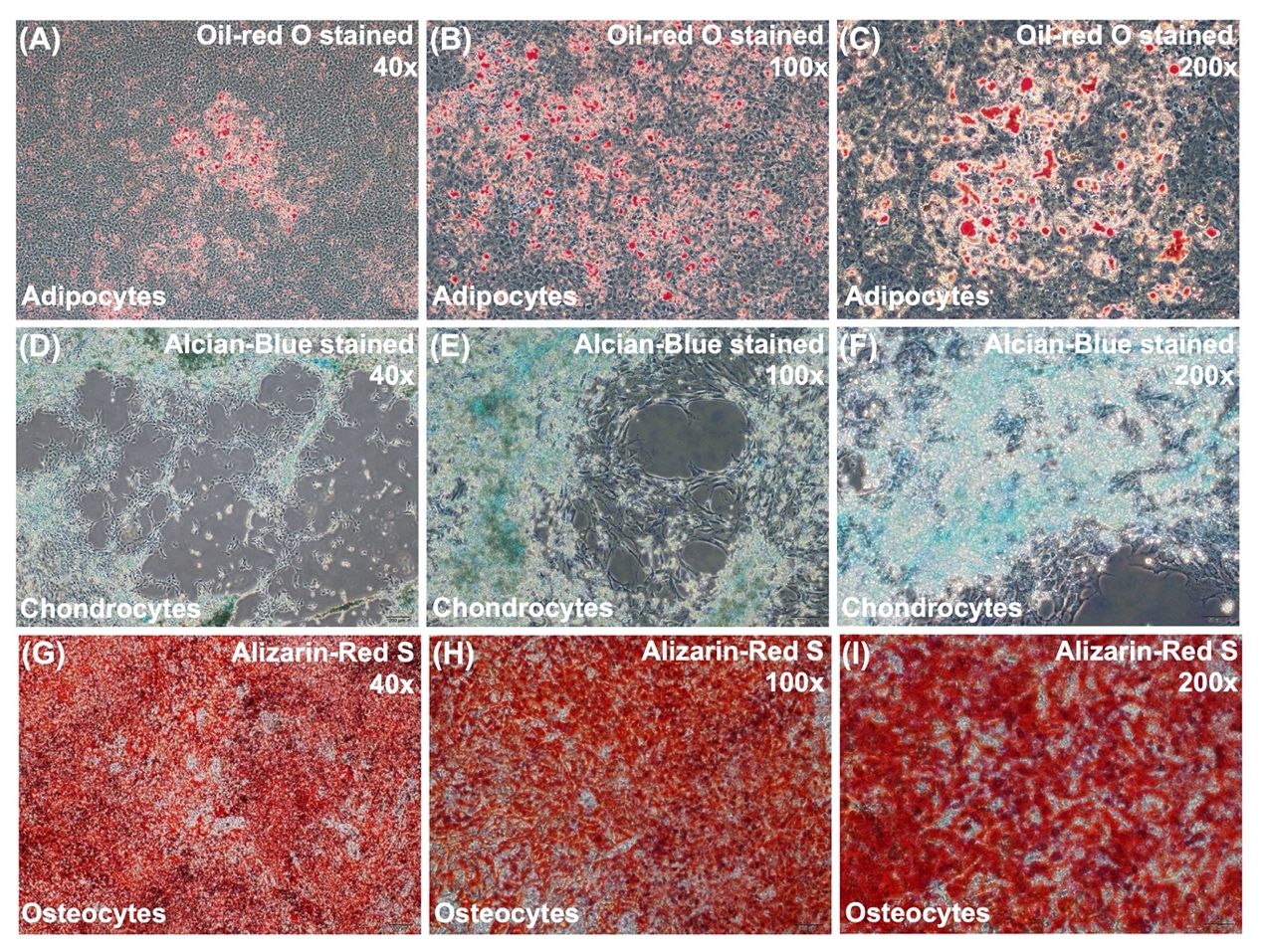
**

**Supplementary Figure 3**


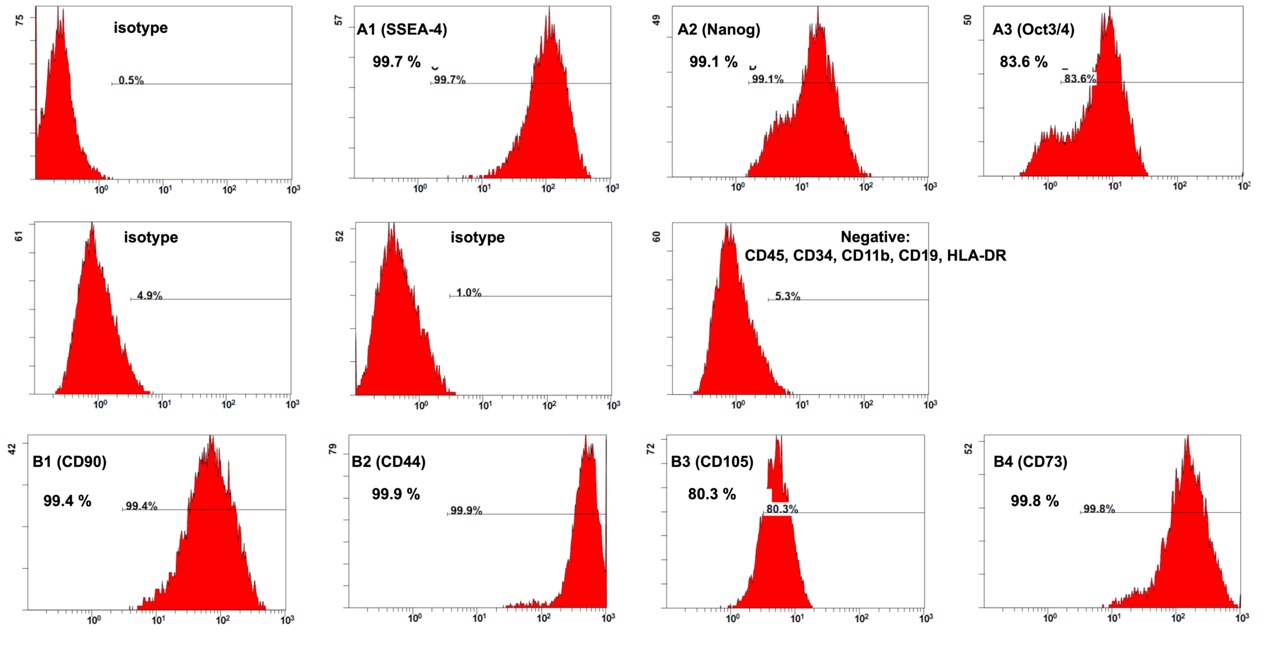

Supplement: Supplementary file 1 — Supplementary Material [file JCMM-25-7675-s001.docx]
